# Unlocking wheat yield potential through evaluation of high-performing hybrid lines in semiarid conditions of northern Pakistan

**DOI:** 10.1371/journal.pone.0356875

**Published:** 2026-09-11

**Authors:** Hamid Ali Khan, Muhammad Arif, Shengquan Zhang, Fazal Munsif, Hongyao Lou, Yutian Gao, Basit Ullah, Muhammad Mehran Anjum, Maaz Khan, Beena Saeed

**Affiliations:** 1 Department of Agronomy, The University of Agriculture, Peshawar, Pakistan; 2 Institute of Hybrid Wheat, Beijing Academy of Agriculture and Forestry Science, Beijing, China; 3 Department of Agronomy, Amir Muhammad Khan Campus Mardan, The University of Agriculture, Peshawar, Pakistan; 4 Department of Agriculture, University of Swabi, Pakhtunkhwa, Pakistan; Jiangsu University, CHINA

## Abstract

Wheat (*Triticum aestivum L*.) yield in Pakistan has remained stagnant over the past two decades mainly due to the narrow genetic diversity of cultivated varieties. To overcome this limitation and explore new genetic resources for improved productivity, twelve Chinese wheat hybrid lines were evaluated in comparison with two local cultivars at the Agronomy Research Farm, the University of Agriculture Peshawar, Pakistan over two seasons (2021–2022, 2022–2023). Results showed that early emergence occurred in the hybrid lines 20 BH 37 and 20 BH 53 (12.5 days). Wheat lines 20 BH 53 and 20 BH 56 produced higher number of tillers m^-2^ (441 and 412), spike density (396 and 391 spikes m^-2^), thousand grain weight (41.9 g and 43.5 g), grains spike^-1^ (54 and 56), biological yield (9561 and 9306 kg ha^-1^), and grain yield (4253 and 4225 kg ha^-1^), respectively. Hybrid lines 20 BH 53 and 20 BH 56 also exhibited a 17–18% increase in grain yield over Wadan-17 and a 16–17% increase over Akbar-19, highlighting their potential as superior genetic resources for wheat productivity enhancement. Positive correlation was observed for grain yield and yield components. Principal component analysis revealed considerable genetic diversity among the tested hybrid lines, supporting their inclusion in future breeding programs. It is concluded that wheat hybrid lines 20 BH 53 and 20 BH 56 outperformed in yield and yield traits over local cultivars under semiarid conditions, indicating their suitability for agro-ecological conditions of northern parts of Pakistan and for future breeding programs.

## Introduction

Wheat (*Triticum aestivum L*.) is a self-pollinated, annual allohexaploid cereal and one of the oldest domesticated members of the Poaceae family [1,2]. Often referred to as the “King of cereals,” wheat is a major global staple cereal crop due to its nutritional value, being rich in carbohydrates, proteins, essential nutrients and dietary fiber [3]. It is the second most widely cultivated cereal crop globally after rice and plays a primary role in food security in Pakistan [4].

In Pakistan, wheat was cultivated on approximately 9 million hectares in the 2022–2023 season, producing 27.63 million tons an increase of 5.4% over the previous year. However, with the country’s population projected to rank sixth globally by 2050, ensuring future food security demands a continued increase in wheat productivity [5]. This challenge is further intensified by climate change which threatens agricultural sustainability.

One of the main constraints to wheat productivity in Pakistan is the limited genetic diversity among stress-resilient and high-yielding varieties [6,7]. Compared to other major wheat-producing nations, Pakistan’s local wheat cultivars show relatively low yield potential. Exploring genetically diverse germplasm is therefore essential to enhance productivity under changing environmental conditions.

To meet the rising demand for wheat, it is essential to identify lines that are not only high-yielding but also well-adapted to local environments [8]. Grain yield is a complex, polygenic trait influenced by genetic makeup, environmental conditions and their interactions [9]. Improvement in yield components such as spike density, grains per spike, and thousand grain weight has been a key breeding strategy [2,10]. Nevertheless, several previously developed varieties failed to perform as expected under field conditions largely due to their poor adaptability to changing climatic conditions [11,12].

Achieving self-sufficiency in wheat requires selecting and cultivating varieties suited to specific agro-climatic zones [13]. Continuous evaluation of promising lines for adaptability and stability is necessary to identify genotypes with desired agronomic traits [14]. Recent breeding efforts using cytoplasmic male sterility (CMS) systems and chemical hybridizing agents have enabled the efficient production of hybrid wheat lines with improved yield, stress tolerance, and disease resistance [15].

Chinese wheat hybrids have recently gained attention for their superior yield potential, especially due to improved spike architecture, grain weight, and biomass production [16]. These hybrids have demonstrated significant variability in phenological and agronomic traits, often surpassing local check varieties in performance [17–19]. Such diversity suggests potential for their use in breeding programs and for direct cultivation under suitable agro-ecological conditions.

While China has made significant advances in hybrid wheat technology, limited studies have evaluated the agronomic performance and genotype × environment (G × E) interactions of these hybrids under Pakistani conditions. Since G × E significantly influences trait expression and yield stability, it is crucial to assess hybrids across varying climatic seasons to understand their adaptability and potential contribution to national food security [19].

This study was conducted to evaluate the yield and yield-related traits of 12 Chinese hybrid wheat lines compared with two local check varieties across two growing seasons in Pakistan. The objective of this study was to evaluate the agronomic and yield performance of hybrid wheat lines across two cropping seasons in order to assess their responsiveness to seasonal variation and to identify promising lines for further evaluation and potential inclusion in future hybrid wheat breeding programs under the agro-ecological conditions of northern Pakistan.

## Materials methods

### Experimental site and characteristics

A field experiment was conducted at Agronomy Research Farm, the University of Agriculture, Peshawar, Pakistan during the winter season 2021–2022 and repeated during winter 2022–2023.

#### Weather conditions.

Weather data for the experimental period (January 2021 to December 2023) were retrieved from NASA Prediction of Worldwide Energy Resources (POWER) database for the coordinates of Agronomy Research Farm, The University of Agriculture, Peshawar (34.0101° N, 71.4833° E; elevation 986.57 m). The dataset included monthly averages of daily minimum, maximum and mean temperatures, along with corrected precipitation (mm/day).

**Fig 1:**
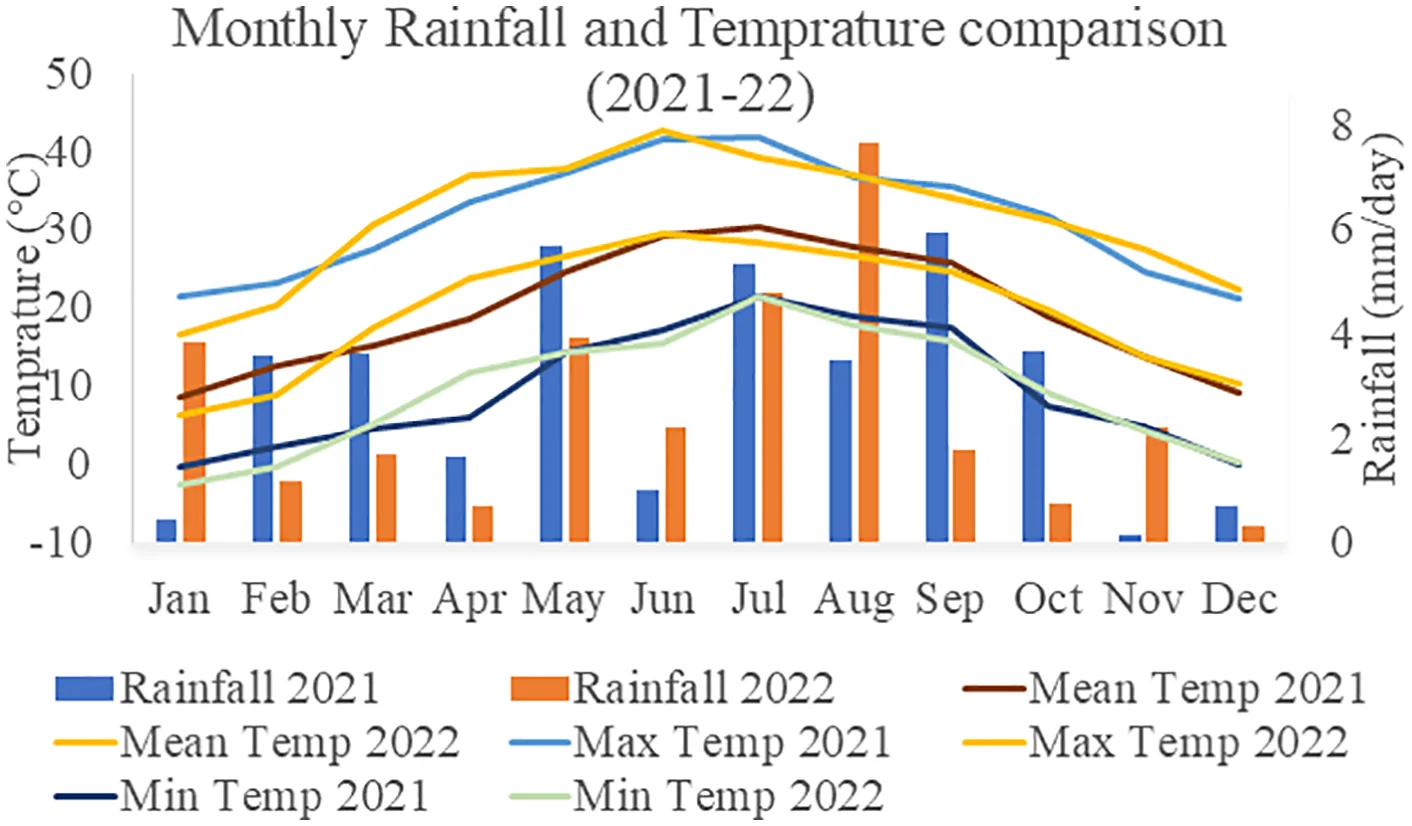
Monthly average temperature (maximum, minimum, and mean) and daily corrected precipitation (mm/day) from January 2021 to December 2022 at the Agronomy Research Farm, the University of Agriculture, Peshawar.

**Fig 2:**
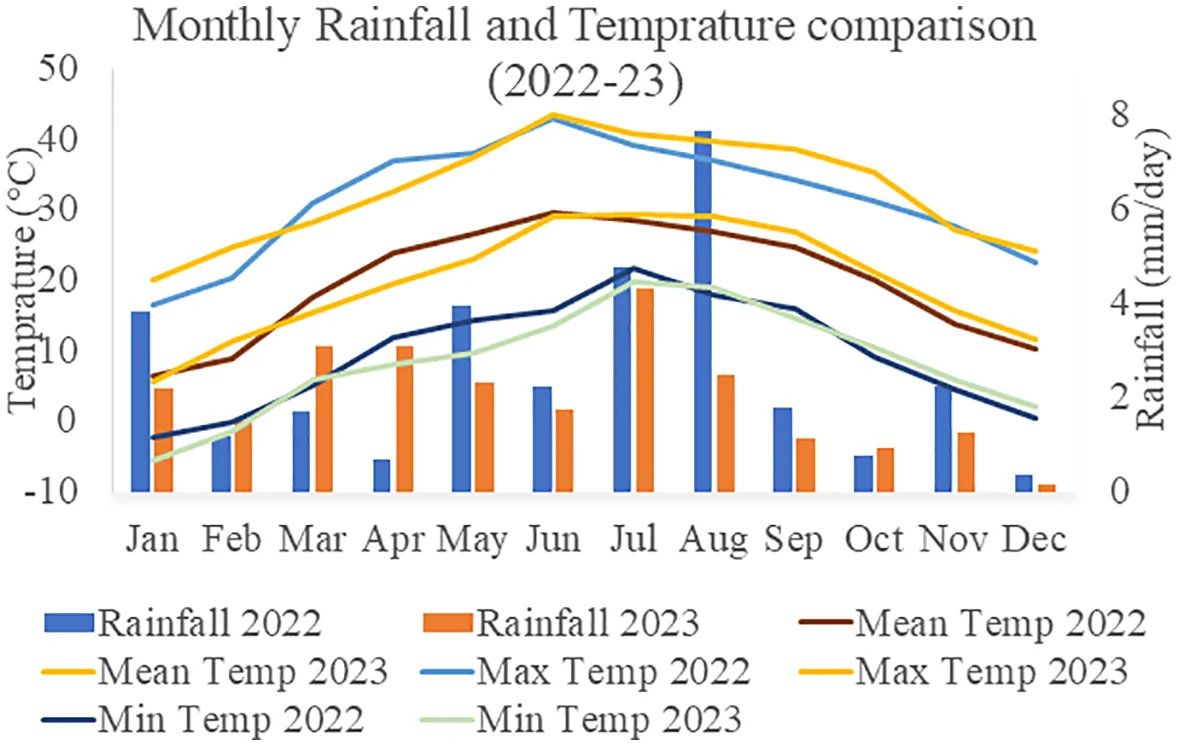
Monthly average temperature (maximum, minimum, and mean) and daily corrected precipitation (mm/day) from January 2022 to December 2023 at the Agronomy Research Farm, the University of Agriculture, Peshawar.

In 2021, the mean annual temperature was 19.58 °C, with the highest maximum in July (41.93 °C) and the lowest minimum in January (−0.26 °C). Precipitation averaged 2.95 mm/day, with peaks in May, July, and September. In 2022, the mean temperature rose slightly to 19.77 °C, while precipitation declined to 2.61 mm/day, with August being the wettest month (7.77 mm/day). The 2023 season maintained a similar mean temperature (19.74 °C), but experienced the highest maximum temperature (43.52 °C in June) and lowest minimum (−5.65 °C in January), with further decline in average rainfall (2.02 mm/day) (Figs 1 and 2).

#### Physicochemical properties of soil.

Physicochemical properties of experimental site are given in (Table 1). A composite soil sample was collected from 0 to 15 cm depth of soil before crop sowing to evaluate the physicochemical properties of soil. The samples were air-dried, finely crushed and passed through a 12 mm sieve prior to analysis. Soil properties analyzed included soil bulk density, texture, pH, electrical conductivity (EC), organic carbon (OC), nitrogen (N), phosphorus (P) and potassium (K). Soil pH and EC were classified according to guidelines of New Mexico State University (NMSU) [20], while soil texture was identified by using Foth method [21]. Kjeldahl apparatus was used to determine the total nitrogen content (%) in the soil [22]. The Olsen extractant sodium bicarbonate method was used to determine the available phosphorus content (mg kg ^−1^) [23], while the AB-DTPA extract method was used to determine the exchangeable potassium [24]. Walkley and Black method was used to determine the soil organic carbon [25]. The soil at the experimental site was silty clay loam textured and had a slightly alkaline pH.

**Table 1:** Physicochemical properties of soil at the experimental site.

Property	Values
**pH**	8
**EC (dS m** ^**−1**^)	0.19
**Sand (%)**	8.68
**Silt (%)**	52.49
**Clay (%)**	38.9
**Textural class**	Silty clay loam
**Bulk Density (Mg m^-3^)**	1.35
**CEC (cmol**_**c**_ **kg**^**-1**^)	30.3
**Total organic C (g kg** ^**−1**^)	12.8
**Available P (mg kg** ^**−1**^)	3.2
**Available N (mg kg**^**-1**^)	22.7
**Available K (mg kg**^**-1**^)	80.8

#### Treatment and field research.

The experiment was performed in Randomized Complete Block Design (RCBD) with wheat lines as the fixed effect being replicated three times during each growing season and was carried out for two consecutive years under similar conditions to evaluate wheat hybrid lines and local cultivars across years. The experimental plot was 2.1 m x 5 m with row spacing of 30 cm accommodating 7 rows. Wheat lines (20 BH 20, 20 BH 23, 20 BH 37, 20 BH 41, 20 BH 45, 20 BH 47, 20 BH 49, 20 BH 52, 20 BH 53, 20 BH 56, 20 BH 57 and 20 BH 66) along with two local varieties Wadan-17 and Akbar-19 were sown at the seed rate of 120 kg ha^-1^ with a hand hoe on 26 November for both years (Y_1_: 2021–2022 and Y_2_: 2022–2023).

#### Agronomic practices.

Prior to the experiment, the field was properly irrigated, tilled at proper moisture condition and levelled using a cultivator up to soil depth of 10–15 cm followed by a rotavator to a depth of 10 cm. Nitrogen and phosphorus were applied at the rate of 120 and 90 kg ha^-1^ from Urea and SSP sources, respectively. Half of nitrogen was applied at time of sowing and the remaining half was applied at heading stage. Phosphorus was applied at the time of sowing. Water for irrigation was used from the Warsak River Canal Peshawar. After sowing, the first irrigation was applied after 20 days, the second after 73 days, and the third irrigation after 112 days for each year. Sulfonet 4% OD (Florasulam 1% w/v 0.98% w/w ± 0.02, Mesosulfuron Methyl 3% w/v 2.94% w/w ± 0.02) was sprayed at a rate of 150 milliliter acre^-1^ to control broadleaf weeds and narrow-leaf (grassy) weeds, respectively. Crop was harvested through sickle when spikes of the wheat crop turned brown. After harvesting, the crop was placed in the field for sun drying for three days for recording further data.

### Crop parameters and analysis

#### Phenological parameters.

Number of days to emergence (DTE) was determined by counting the days from the sowing date until 75% visible emergence of seedlings above the soil surface were seen. Days to physiological maturity (PM) were recorded from sowing to the stage when the majority of plants exhibited yellowing of leaves and stems, signaling the end of nutrient translocation.

#### Growth parameters.

Data concerning tillers m^-2^ (TIL) was determined by taking three rows of 1 meter length randomly in each plot and number of tillers were counted and then it was converted to tillers m^-2^. Data regarding spike weight (g) was recorded by weighing ten spikes in each row by digital balance and then it was averaged.

#### Yield and yield components.

Spikes m^-2^ (Spm) were recorded by counting the number of spikes within a 1-meter length of three randomly selected rows per plot and then converted to spikes per square meter. The number of grains per spike (GPS) was determined by randomly selecting five spikes from each experimental unit. The grain count for these spikes was recorded and averaged, providing an estimate of grains per spike. Thousand grain weight (TGW) was measured by counting 1000 grains from each plot and weighing them using a digital balance. Biological yield (BY) was measured by harvesting above-ground biomass of three central rows in each plot. It was then weighed by digital balance and converted to kg ha^-1^. Grain yield (GY) was measured by harvesting three central rows of each experimental unit and then it was threshed by mini thresher. Weighing was done with the help of digital balance and data was converted to kg ha^-1^. Data on harvest index (HI) was calculated as the determining ratio of grain yield to biological yield.

#### Statistical analysis.

Before statistical analysis, the data collected for both years were tested initially for normality using Shapiro–Wilk test and homogeneity of variances by Levene’s test using R software. Following assumptions for normality tests, the data were subjected to further analysis using Statistix 8.1 software. Statistical analysis was performed combined over years using procedure as outlined by [26]. Year treated as random effect while wheat lines as fixed effect. The analysis revealed partitioning of total variance into components due to lines, years, and their interaction. Multiple mean comparison test (LSD) was used for significant differences among the lines means at 5% level of probability. Biplot and correlation analysis were performed using statistical software R (4.2.1) to evaluate association among the measured traits and identify key contributing variables. Moreover, Hierarchical Cluster Analysis (HCA) was carried out for classification of tested lines into distinct clusters based on agronomic attributes. Significance levels are indicated as P < 0.05 (*), P < 0.01 (**), and NS denotes non-significant differences. Different letters next to means indicate statistically significant differences based on LSD at 5% probability.

## Results

### Phenological parameters of wheat lines

Wheat hybrid lines differed significantly for days to emergence and physiological maturity during both years (Table 2). Early emergence occurred in wheat lines 20 BH 37, 20 BH 53 (12.5 days) and Wadan-17 (12.6 days). Late emergence was recorded for 20 BH 56 (15.1 days) which was statistically similar to 20 BH 45, 20 BH 41, 20 BH 23, 20 BH 47, 20 BH 49, and 20 BH 57. A significant year effect was noted with mean emergence occurring earlier in 2021−2022 (13.1 days) than in 2022−2023 (14.0 days). Similarly, significant differences were found among hybrid lines for physiological maturity. The early maturing lines were 20 BH 47 and 20 BH 23 (145.8 and 146.6 days, respectively), while the late maturity was observed in Akbar-19 (151.1 days) which was statistically at par with Wadan-17 (150.6) 20 BH 66 (150.6), 20 BH 20 (149.5), 20BH 53 (149.5), 20 BH 56 (149.3) and 20 BH 57 (148.8). The effect of year was not significant for physiological maturity. Similarly, the genotype × year interaction was also not significant for physiological maturity.

**Table 2:** Days to emergence (DTE) and physiological maturity (PM) of wheat lines evaluated during winter seasons 2021-2022 and 2022-2023.

Lines	Days to emergence		Means	Physiological maturity		Means
	2021-2022	2022-2023		2022-2023	2021-2022	
**20 BH 20**	13.3	12.3	12.8^de^	149.6	149.3	149.5^abcd^
**20 BH 23**	15.0	14.3	14.6^abcd^	147.3	146.0	146.6^f^
**20 BH 37**	11.3	13.6	12.5^e^	146.6	146.0	147.5^def^
**20 BH 41**	14.6	15.0	14.8^a-d^	145.3	149.0	147.1^def^
**20 BH 45**	15.0	15.0	15^ab^	146.0	148.0	147^def^
**20 BH 47**	13.0	14.6	13.8^a-e^	145.6	146.0	145.8^f^
**20 BH 49**	14.6	12.3	13.5^a-e^	148.0	148.3	148.1^b-f^
**20 BH 52**	12.6	13.3	13^cde^	146.3	149.6	148.0^c-f^
**20 BH 53**	11.3	13.6	12.5^e^	149.0	150.0	149.5^a-d^
**20 BH 56**	14.3	16.0	15.1^a^	149.0	149.6	149.3^a-e^
**20 BH 57**	13.6	14.3	14^a-e^	149.3	148.3	148.8^a-e^
**20 BH 66**	12.3	13.6	13^cde^	151.0	150.3	150.6^abc^
**Wadan-17**	11.0	14.3	12.6^e^	149.6	152.0	150.6^abc^
**Akbar-19**	12.0	14.3	13.1^b-e^	151.0	151.3	151.1^a^
**Means**	13.1^b^	14.0^a^		148.1^a^	149.0^a^	
Source of Variation		Significance of F test				
Lines (L)		*	*			
Years (Y)		**	NS			
(L xY)		NS	NS			

*, ** Significant at 5% and 1% level of probability, NS: non-significant. Based on LSD test, means in the same columns followed by different letters differed significantly according to LSD test (p ≤ 0.05).

### Growth parameters of wheat lines

Data concerning tillers m^-2^ of wheat lines are given in (Table 3). Data analysis revealed that wheat hybrid lines varied significantly for tillers m^-2^. Mean data of the hybrid lines exhibited that higher tillers (441 and 412 m^-2^) were produced by 20 BH 53 and 20 BH 56, respectively followed by 20 BH 57 which produced 405 tillers m^-2^. Minimum tillers (313 m^-2^) were recorded for 20 BH 45. The year effect was not significant. Likewise, the G xY interaction was also not significant.

**Table 3:** Tillers m^-2^ of wheat lines evaluated under semiarid conditions during winter seasons 2021-2022 and 2022-2023.

Lines	Tillers m^-2^		Means
	2021-2022	2022-2023	
**20 BH 20**	324	333.3	329^fg^
**20 BH 23**	352.3	359	356^def^
**20 BH 37**	345.7	347.7	347^ef^
**20 BH 41**	373.3	359	366^de^
**20 BH 45**	314.7	312	313^g^
**20 BH 47**	352	343	348^ef^
**20 BH 49**	347.3	360	354^def^
**20 BH 52**	391	382.3	382^cd^
**20 BH 53**	452.7	429.3	441^a^
**20 BH 57**	409.3	400.7	405^bc^
**20 BH 66**	384	370	377^cd^
**Wadan-17**	371.3	386	379^cd^
**Akbar-19**	342.7	374	358^de^
**Means**	367.6^a^	370.1^a^	
Source of Variation	Significance of F test		
Lines (L)	*		
Years (Y)	NS		
(L x Y)	NS		

*, ** Significant at 5% and1% level of probability, NS: non-significant. Based on LSD test, means in the same columns followed by different letters differed significantly according to LSD test (p ≤ 0.05).

### Yield and yield components of wheat lines

Wheat hybrid lines varied significantly for spikes m^-2^, spike weight, grains spike^-1^ and thousand grain weight. The year as source of variation had no significant effect on these parameters. The interaction between lines × year was also not significant for these parameters. Spikes m^-2^ ranged between 295.3 to 396.0 with the highest value for 20 BH 53 (396 spikes m^-2^) which was statistically at par with hybrid lines 20 BH 56 (391 m^-2^) and 20 BH 57 (381.6 m^-2^) followed by wheat line 20 BH 52 (361.6 m^-2^)**,** whereas lower number of spikes m^-2^ was recorded for wheat hybrid line 20 BH 45 (295.3 m^-2^)**.** Spike weight was greater for hybrid line 20 BH 56 (4.4 g) and was statistically similar to 20 BH 53 (4.1 g)**,** Akbar-19 (4.18 g)**,** Wadan-17 (4.0 g)**,** 20 BH 66 (4.0 g)**,** and 20 BH 57 (3.9 g)**.** Whereas, the lowest spike weight (3.3 g) was noted for hybrid line 20 BH 20 (Table 4).

**Table 4:** Spikes m^-2^ and spike weight (g) of wheat lines evaluated under semiarid conditions during winter seasons 2021-2022 and 2022-2023.

Lines	Spikes m^-2^		Means	Spike weight (g)		Means
	2021-2022	2022-2023		2021-2022	2022-2023	
**20 BH 20**	308.3	314	311.1^gh^	3.26	3.43	3.3^e^
**20 BH 23**	304	343	323.5^fgh^	3.73	3.56	3.65^de^
**20 BH 37**	329.7	338	333.8^d-g^	3.76	3.6	3.68^cde^
**20 BH 41**	348	346	347.0^d-g^	3.66	3.63	3.6^de^
**20 BH 45**	297.7	293	295.3^h^	3.83	3.6	3.7^b-e^
**20 BH 47**	331	321.7	326.3^e-h^	3.6	3.8	3.7^cde^
**20 BH 49**	331	337.3	334.1^d-g^	3.76	3.56	3.66^de^
**20 BH 52**	356.3	367	361.6^bcd^	3.36	3.9	3.63^de^
**20 BH 53**	383	409	396^a^	4.13	4.16	4.1^abc^
**20 BH 56**	373.3	408.7	391^ab^	4.54	4.26	4.4^a^
**20 BH 57**	385.7	377.7	381.6^abc^	4.03	3.83	3.9^a-d^
**20 BH 66**	363.7	349.3	356.5^cde^	4	4.16	4^a-d^
**Wadan-17**	349.7	364.3	357^cde^	3.7	4.3	4^a-d^
**Akbar-19**	327.7	354.3	341^d-g^	4.03	4.33	4.18^ab^
**Means**	342.1^a^	351.6^a^		3.81^a^	3.87^a^	
Source of Variation	Significance of F test					
Lines (L)	**	**				
Years (Y)	NS	NS				
(L x Y)	NS	NS				

*, ** Significant at 5%,1% level of probability, NS: non-significant. Based on LSD test, means in the same columns followed by different letters differed significantly according to LSD test (p ≤ 0.05).

Grains spike^-1^ ranged from 38 to 56 grains among the evaluated lines. More grains per spike (56 grains) were recorded for 20 BH 56. Less number of grains per spike (38 grains) were observed for 20 BH 45. Compared with local cultivars, hybrid lines 20 BH 56 and 20 BH 53 showed increases of 17% and 13% over Wadan-17, 14% and 10% grains per spike over Akbar-19, respectively. Moreover, hybrid lines 20 BH 66 and 20 BH 52 also showed comparable increase in grains number over local checks whereas the hybrid line 20 BH 45 produced lower number of grains spike^-1^ (Table 5). The heavier grains based on thousand grain weight data were recorded for hybrid lines 20 BH 56 (43.5 g) and 20 BH 53 (41.9 g) indicating an increase of 8 and 4% over local check (Wadan-17), respectively and 6 and 2% increase over 2^nd^ local check (Akbar-19), respectively. The lowest thousand grain weight was obtained by hybrid lines 20 BH 20 (37.0 g) and 20 BH 45 (36.7 g).

**Table 5:** Grains spike^-1^ (GPS) and thousand grain weight (g) (TGW) of wheat lines evaluated under semiarid conditions during winter seasons 2021-2022 and 2022-2023.

Lines	GPS		Means	TGW (g)		Means
	2021-2022	2022-2023		2021-2022	2022-2023	
**20 BH 20**	44	45	45^cdef^	34.4	37.5	37.0^f^
**20 BH 23**	48	43	46^bcde^	37.2	34.2	36.7^f^
**20 BH 37**	48	41	44^cdef^	38	37.4	37.7^ef^
**20 BH 41**	45	46	45^cdef^	38.1	37.3	37.7^def^
**20 BH 45**	39	38	38^g^	36.1	37.3	36.7^f^
**20 BH 47**	44	43	43^def^	39.7	38.8	39.2^b-f^
**20 BH 49**	43	42	43^ef^	39.7	43.4	41.0^abc^
**20 BH 52**	44	40	42^fg^	38	39.3	38.6^cdef^
**20 BH 53**	55	53	54^a^	39.9	44.1	41.9^ab^
**20 BH 56**	56	56	56^a^	41.7	45.4	43.5^a^
**20 BH 57**	44	43	44^def^	36.7	39.3	37.9^cdef^
**20 BH 66**	45	48	47^bcd^	38.3	39.4	38.8^b-f^
**Wadan-17**	48	48	48^bcd^	40.3	40.2	40.2^bcde^
**Akbar-19**	48	50	49^bc^	40.6	41.2	40.9^abcd^
**Means**	46^a^	45^a^		38.6^b^	39.8^a^	
Source of Variation			Significance of F test			
Lines (L)			**	**		
Years (Y)			NS	*		
(L x Y)			NS	NS		

*, ** Significant at 5%,1% level of probability, NS: non-significant. Based on LSD test, means in the same columns followed by different letters differed significantly according to LSD test (p ≤ 0.05).

### Biological yield, grain yield and harvest index

Biological yield, grain yield and harvest index were significantly affected by wheat hybrid lines. Year as a source of variation had no significant effect on these parameters. The interaction between years and lines were found non-significant for biological yield, grain yield and harvest index (Table 6 and 7). Higher biological yields of 9561 and 9306 kg ha^-1^ were produced by 20 BH 53 and 20 BH 56, respectively which were statistically similar with Akbar-19 and 20 BH 49 (9162 and 9059 kg ha^-1^), respectively. Compared to local cultivars, the hybrid lines 20 BH 53 and 20 BH 56 produced 9 and 6% higher biological yield than local checks Wadan-17 and 4 and 1.5% over Akbar-19, respectively. The lowest biological yields (7948, 7888, 7848, 7833 and 7760 kg ha^-1^) were produced by 20 BH 45, 20 BH 37, 20 BH 20, 20 BH 41 and 20 BH 23, respectively. Maximum grain yields (4253 and 4225 kg ha^-1^) were produced by 20 BH 53 and 20 BH 56 followed by Akbar-19 and 20 BH 52 (3644 and 3642 kg ha^-1^). This represents a substantial increase of about 15 and 14% in yield for the top two hybrid lines (20 BH 53 and 20 BH 56) over the local checks Wadan-17, and Akbar-19, respectively indicating the superiority of the exotic hybrid lines. Minimum grain yields (3294 and 3287 kg ha^-1^) were produced by 20 BH 23 and 20 BH 37. In case of years, higher grain yield (3484 kg ha^-1^) was recorded during first year followed by second year (3613 kg ha^-1^). Harvest index was the highest for 20 BH 56 (45.3%) followed by 20 BH 53 (44.8%) which was statistically at par with 20 BH 41, 20 BH 66, 20 BH 52, 20 BH 20, 20 BH 23, 20 BH 45, 20 BH 57 and 20 BH 37 (43.8, 43.8, 43.6, 42.6, 42.6, 42.1, 42.1 and 41.8), respectively. Lower HI was measured for 20 BH 47 (41.6%) and Akbar-19 (40.3%).

**Table 6:** Biological yield (kg ha^-1^) and grain yield (kg ha^-1^) of wheat lines evaluated under semiarid condition during winter seasons 2021-2022 and 2022-2023.

Lines	Biological yield (kg ha^-1^)		Means	Grain yield (kg ha^-1^)		Means
	2021-2022	2022-2023		2021-2022	2022-2023	
**20 BH 20**	7622	8074	7848^e^	3421	3230	3325^de^
**20 BH 23**	7704	7816	7760^e^	3278	3311	3294^e^
**20 BH 37**	7862	7914	7887^e^	3299	3275	3287^e^
**20 BH 41**	7781	7914	7832^e^	3348	3524	3436^b-e^
**20 BH 45**	7944	7951	7947^e^	3234	3448	3341^cde^
**20 BH 47**	8310	8338	8323^de^	3374	3443	3408^b-e^
**20 BH 49**	8826	9293	9059^abc^	3410	3224	3317^de^
**20 BH 52**	8170	8587	8378^cde^	3410	3874	3642^b^
**20 BH 53**	10049	9074	9561^a^	4262	4244	4253^a^
**20 BH 56**	9254	9358	9306^ab^	4374	4077	4225^a^
**20 BH 57**	7699	8105	7902^e^	3238	3396	3317^de^
**20 BH 66**	7817	8492	8154^de^	3274	3873	3573^b-e^
**Wadan-17**	8773	8670	8721^bcd^	3581	3640	3610^bc^
**Akbar-19**	9628	8697	9162^ab^	3566	3723	3644^b^
**Means**	8388^a^	8446^a^		3484^b^	3613^a^	
Source of Variation		Significance of F test				
Lines (L)		**	**			
Years (Y)		NS	*			
(L x Y)		NS	NS			

*, ** Significant at 5%,1% level of probability, NS: non-significant. Based on LSD test, means in the same columns followed by different letters differed significantly according to LSD test (p ≤ 0.05).

**Table 7:** Harvest index (%) of wheat lines evaluated under semi-arid conditions during winter seasons 2021-2022 and 2022-2023.

Lines	Harvest index (%)		Means
	2021-2022	2022-2023	
**20 BH 20**	45	40.3	42.6^abc^
**20 BH 23**	42.7	42.7	42.6^abc^
**20 BH 37**	42	41.7	41.8^abc^
**20 BH 41**	43	44.7	43.8^abc^
**20 BH 45**	41	43.3	42.1^abc^
**20 BH 47**	41	41.3	41.1^c^
**20 BH 49**	38.7	35	36.8^d^
**20 BH 52**	42.3	45	43.6^abc^
**20 BH 53**	42.7	47	44.8^ab^
**20 BH 56**	44	46.7	45.3^a^
**20 BH 57**	42.3	42	42.1^abc^
**20 BH 66**	42	45.7	43.8^abc^
**Wadan-17**	41	42.3	41.6^bc^
**Akbar-19**	37.3	43.3	40.3 cd
**Means**	41.8^a^	42.9^a^	
Source of Variation	Significance of F test		
Lines (L)		**	
Years (Y)		NS	
(L x Y)		NS	

*, ** Significant at 5%,1% level of probability, NS: non-significant. Based on LSD test, means in the same columns followed by different letters differed significantly according to LSD test (p ≤ 0.05).

### Correlation (r)

**Fig 3:**
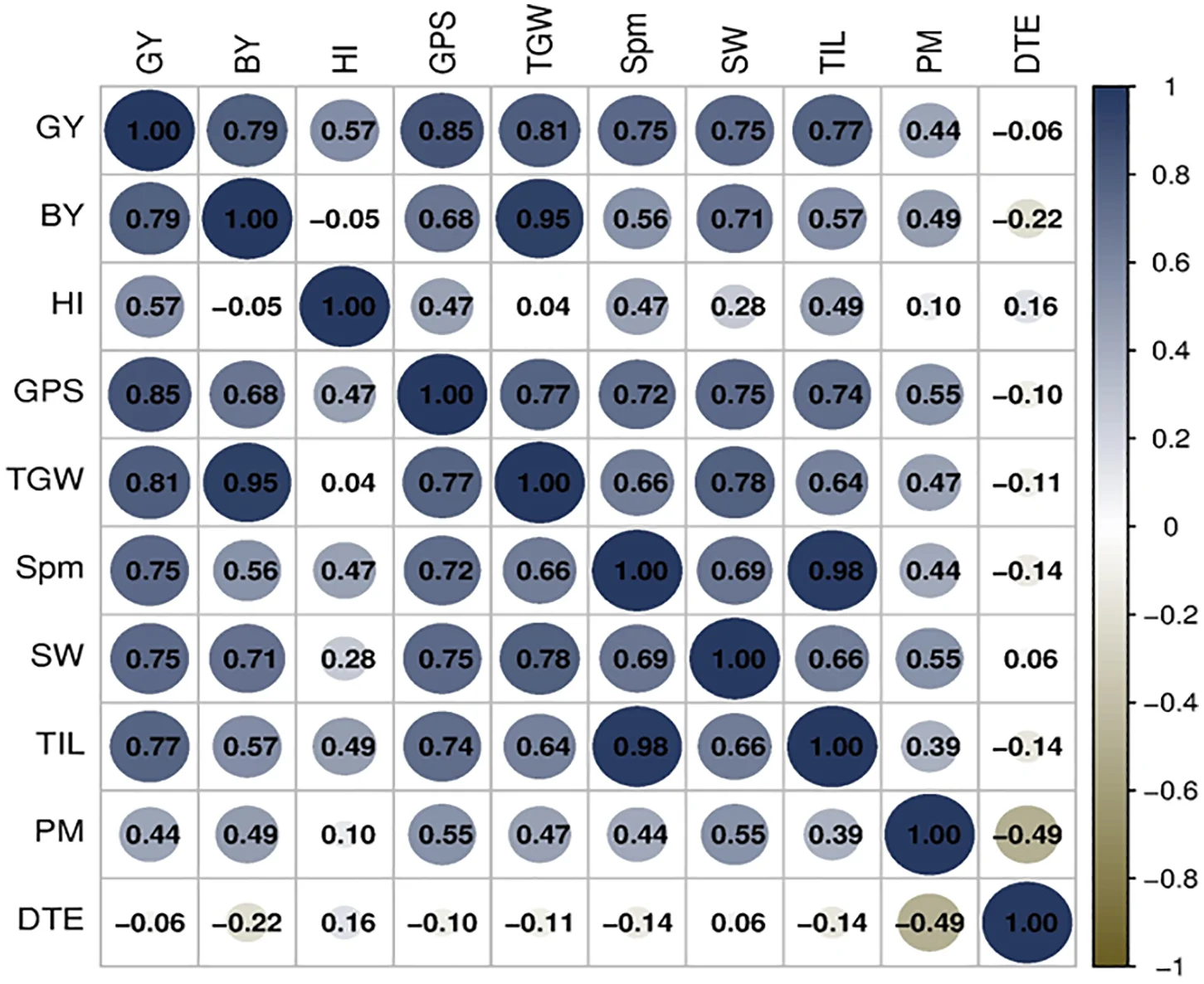
Pearson’s correlation coefficients (r) among agronomic traits of wheat hybrid lines evaluated under semiarid conditions during two growing seasons. DTE (days to emergence), PM (Physiological maturity), TIL (Tillers m^-2^), Spm (Spikes m^-2^), SW (Spike weight), GPS (grains spike^-1^), TGW (thousand grain yield), BY (Biological yield), GY (Grain yield) and HI (Harvest index).

The Pearson’s correlation illustrates the relationships between various agronomic traits (Fig 3). The color gradient, ranging from blue to red, represents the strength and direction of the correlation coefficients, with red indicating a strong positive correlation and blue indicating a strong negative correlation. Grain yield shows strong positive correlations with grains spike^-1^ (r = 0.85), spike weight (r = 0.76), and biological yield (r = 0.81), indicating that increase in grains spike^-1^, spike weight, and biological yield are significantly associated with grain yield. Similarly, thousand grain weight has a very strong positive correlation with grain yield (r = 0.95) and biological yield (r = 0.95). Negative correlations are also evident, such as plant maturity being negatively correlated with days to emergence (r = −0.5), suggesting that earlier emergence tends to lead to later plant maturity. Additionally, biological yield shows a moderate negative correlation with days to emergence (r = −0.22).

### Principal component analysis (PCA)

**Fig 4:**
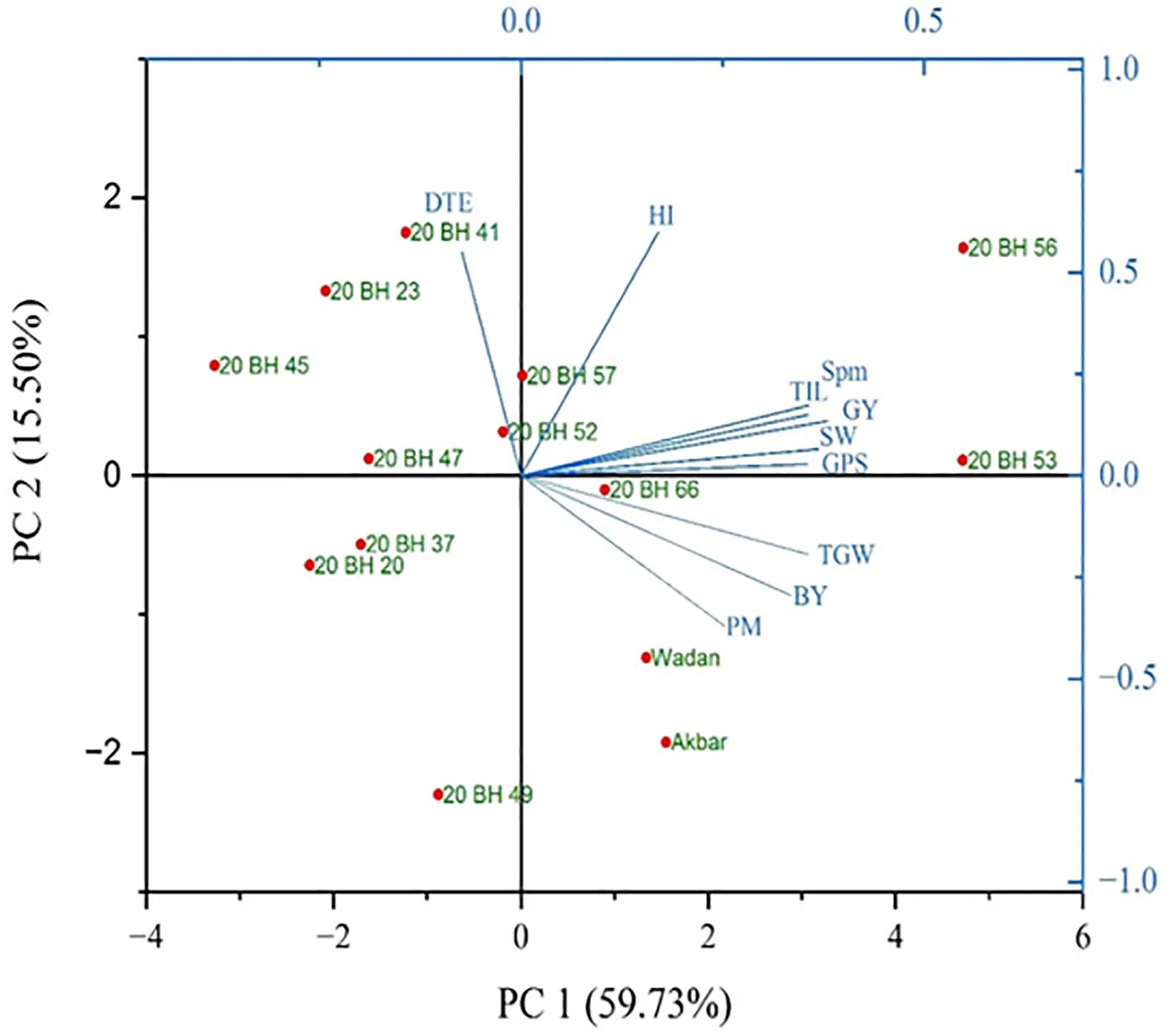
Principal component analysis (PCA) biplot illustrating relationships among agronomic traits and wheat hybrid lines under semiarid conditions during two growing seasons. DTE (days to emergence), PM (Physiological maturity), TIL (Tillers m^-2^), Spm (Spikes m^-2^), SW (Spike weight), GPS (grains spike^-1^), TGW (thousand grain yield), BY (Biological yield), GY (Grain yield) and HI (Harvest index).

The PCA biplot provides valuable insights into the relationships among agronomic traits and the distribution of wheat hybrid lines, aiding in the selection of lines with desirable trait combinations. The PCA biplot revealed the distribution and relationships between various agronomic traits and wheat hybrid lines, with PC1 explaining 59.73% and PC2 explaining 15.50% of the total variation (Fig 4). Traits such as grain yield, spike weight, grains spike^-1^, spikes m^-2^, thousand grain weight and biological yield are positively correlated with PC1, indicating their significant contribution to the variation captured by PC1. Days to emergence and harvest index are positively correlated with PC2, suggesting their contribution to the variation captured by PC2. Wheat hybrid lines 20 BH 56 and 20 BH 53 are strongly associated with traits positively correlated with PC1, while Wadan-17 and Akbar-19 are associated with traits positively correlated with PC2. The close grouping of traits like GY, SW, GPS, and TGW indicated a strong positive correlation among these traits.

### Hierarchical clustering of wheat hybrid lines

**Fig 5:**
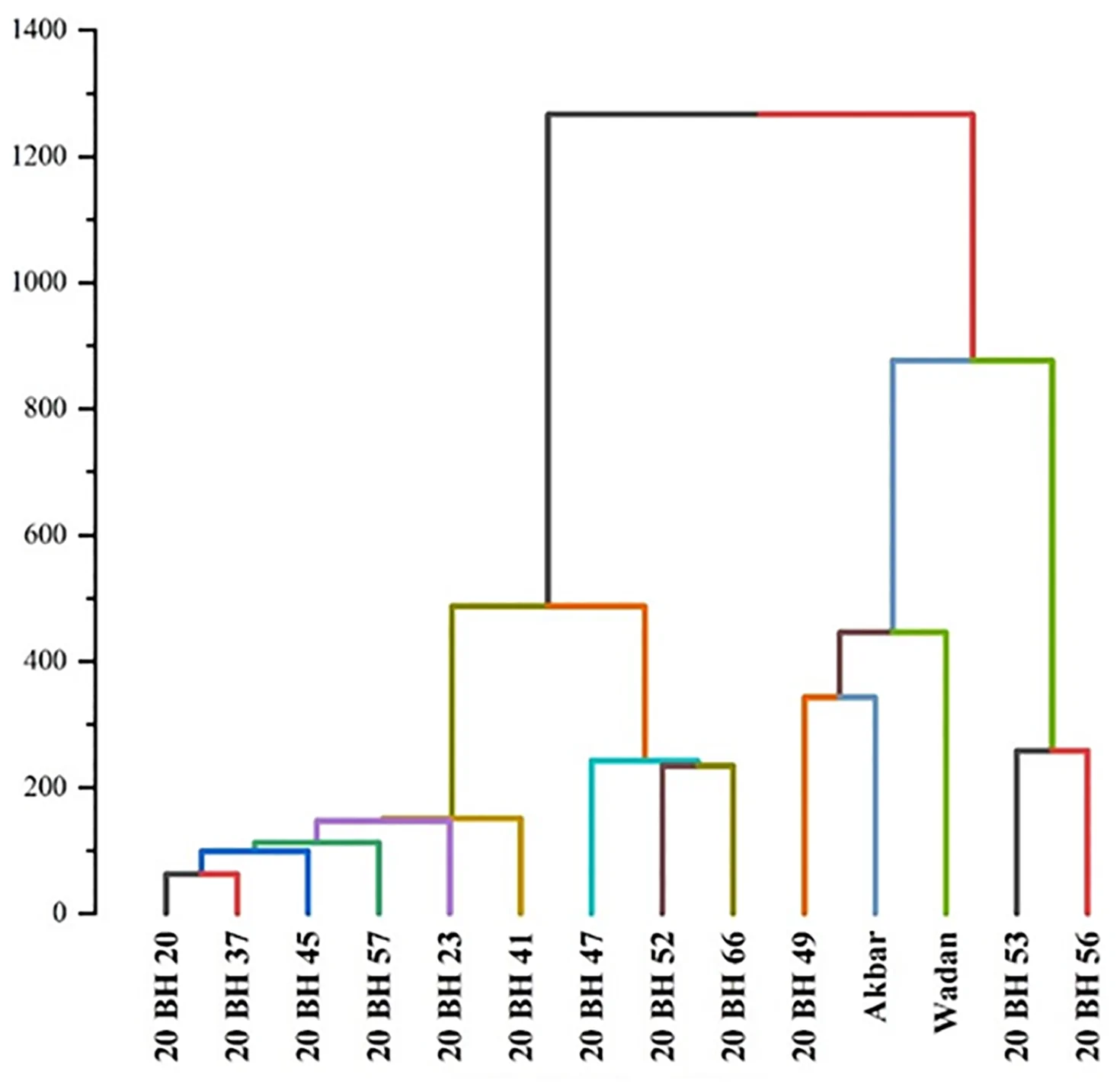
Hierarchical cluster dendrogram showing genetic similarity among wheat lines evaluated under semiarid conditions during two seasons.

The dendrogram illustrates the hierarchical clustering of wheat hybrid lines based on their agronomic traits, revealing two major clusters (Fig 5). The first major cluster includes the wheat hybrid lines 20 BH 20, 20 BH 37, 20 BH 45, 20 BH 57, 20 BH 23, 20 BH 41, 20 BH 47, 20 BH 52, and 20 BH 66. Within this cluster 20 BH 20 and 20 BH 37 form a close sub-cluster, indicating high similarity. Similarly, 20 BH 41 and 20 BH 47 are closely related. The second major cluster comprises 20 BH 49, Akbar-19, Wadan-17, 20 BH 53 and 20 BH 56. Within this group, Akbar-19 and Wadan-17 form a distinct sub-cluster, as do 20 BH 53 and 20 BH 56. Notably, 20 BH 20 and 20 BH 37 are the most similar lines, while 20 BH 49 and 20 BH 56 exhibit the greatest dissimilarity, indicating significant diversity in their traits. Overall, the dendrogram effectively categorizes the wheat hybrid lines, providing insights into their genetic relationships and potential for breeding programs.

## Discussion

The inertia of wheat yield in Pakistan, a crucial challenge for national food security, is mainly ascribed to the narrow genetic base of prevailing cultivars, which curtail their potential for adaptation and improvement under the changing climatic conditions [27,38]. This study establishes that introducing genetically diverse hybrid wheat lines from China presents a workable strategy to halt this genetic bottleneck. Our evaluation of twelve hybrid lines in comparison to the two local checks under semi-arid conditions resulted in significant genotypic variation across all measured phenological, growth, and yield parameters. The superior and steady performance of hybrids 20 BH 53 and 20 BH 56 across two climatically distinct seasons. It underlines their potential as superior inherited resources for immediate use and future breeding in Pakistan [16,28].

The observed variation in phenological traits, viz. days to emergence and physiological maturity, revealed the diverse genetic backgrounds and environmental sensitivities of the tested hybrid lines. The early emergence of lines such as 20 BH 37 and 20 BH 53 advocates expedient seed robustness and swift establishment, which can be vital for weed competition and early resource acquisition in variable environments [29,30]. In contrast, the later emergence of 20 BH 56 did not reduce its final yield, indicating compensatory mechanisms during succeeding growth phases, a flexibility trait increasingly recognized in modern cultivars [31]. The crucial effect of the year on emergence time, with later mean emergence in the warmer, drier second year, directly highlights the stimulus of climatic variability on this initial developmental stage, consistent with findings on thermal-time requirements for germination [32]. For physiological maturity, the range observed demonstrates genetic differences in the duration of the reproductive cycle [33]. The high-yielding hybrids 20 BH 53 and 20 BH 56 showed an optimum maturity period that balanced sufficient time for grain filling with dodging of terminal heat stress, a key adaptive feature for variable environments targeted in contemporary breeding [34,35].

Among growth parameters, the significant differences in tiller density m^-2^ are essential indicator of yield potential. Hybrids 20 BH 53 and 20 BH 56 produced the highest number of tillers, an inbred trait associated with vigorous vegetative growth and effective resource uptake during the tillering phase, which is a principal determinant of final spikes number [35,36]. This vigorous initial growth directly supported their succeeding superior performance [37].

The examination of yield components provides a clear explanation for differences in final grain. The elite hybrids surpassed in a synergistic manner across numerous components. Firstly, they produced an expressively greater number of spikes m^-2^, translating their high tillers count into a high number of fertile, grain-bearing structures, a direct contributor to yield in resource-limited environments [38,39]. Secondly, they achieved superior spike weight, demonstrating a greater biomass allocation to each reproductive unit. Thirdly, and most remarkably, they displayed a remarkable increase in the number of grains per spike and thousand-grain weight. The considerable increases in grains per spike over the local checks point to enhanced spike fertility and floret endurance, traits administered by genetic potential and effective carbon partitioning during critical developmental windows, a key focus of physiological breeding [38,39]. Simultaneously, the higher thousand-grain weight demonstrates superior grain-filling capacity and starch deposition, linked to constant photosynthetic activity and efficient remobilization of stem reserves under semi-arid conditions [40,41]. This synchronized improvement in spike density, prolificacy, and grain size is a typical manifestation of heterosis and was the direct driver of the high grain yield [42].

The final yield limitations, biological yield and harvest index, depicts the physiological picture. Hybrids 20 BH 53 and 20 BH 56 achieved the highest biological yield, revealing their overall vigorous photosynthetic ability and total biomass production. Decisively, they also maintained a high harvest index, which reflects the efficient partitioning of this acquired biomass into the economic yield, the grain. This balance is crucial; a high biological yield without a correspondingly high harvest index does not translate to superior grain production [43,44]. The aptitude of these hybrids to surpass in both metrics underscores their breeding value for achieving both high productivity and efficient resource alteration.

The association and multivariate analyses statistically authenticate the relationships inferred from the mean data. The strong positive correlations between grain yield and components like grains per spike and thousand-grain weight confirm that improvements in these traits were directly linked with the final yield advantage, a pattern consistently reported in genotype evaluations under stress [45]. The principal component analysis evidently separated the elite hybrids from the local checks along the principal alliance, which was heavily loaded with yield and yield-component traits, providing a pictorial representation of their superior and distinct phenotypic profile, a useful tool for genotype selection [46].

A fundamental conclusion was the performance stability of the top hybrids. Despite the second season being prevailed by higher temperatures and lower rainfall, 20 BH 53 and 20 BH 56 sustained their high yield. The general absence of significant genotype-by-year relations for most components indicates unchanging trait expression under these fluctuating conditions. This resilience recommends an inherent tolerance to abiotic stresses like heat and moisture shortfall, a critical attribute for ensuring yield stability in the face of climate unpredictability, a principal breeding objective.

## Conclusion

In conclusion, this comprehensive evaluation reveals that Chinese hybrid wheat lines, predominantly 20 BH 53 and 20 BH 56, own a superior and well-integrated set of agronomic traits. Their significant yield advantage over local cultivars is built upon a foundation of dynamic growth, leading to high tiller and spike density, which is combined with extraordinary spike fertility, grain size, and efficient biomass accumulation. Their reliable performance across contrasting seasons further recommends them for practical agricultural use. Simultaneously, their favorable genetic attributes should be introgressed into local breeding programs to augment the genetic base and catalyze the development of next-generation, high-yielding, and climate-resilient wheat varieties for Pakistan.

## Supporting information

S1 File Checklist global research questionnaire. (DOCX)

S2 File Two years data with ANoVA and mean tables. (XLSX)
